# OneHealth Approaches Contribute Towards Antimicrobial Resistance: Malaysian Perspective

**DOI:** 10.3389/fmicb.2021.718774

**Published:** 2021-10-25

**Authors:** Vanitha Mariappan, Kumutha Malar Vellasamy, Nor Alia Mohamad, Sreeramanan Subramaniam, Jamuna Vadivelu

**Affiliations:** ^1^Centre of Toxicology and Health Risk Studies (CORE), Faculty of Health Sciences, Universiti Kebangsaan Malaysia, Kuala Lumpur, Malaysia; ^2^Department of Medical Microbiology, Faculty of Medicine, University of Malaya, Kuala Lumpur, Malaysia; ^3^Biomedical Science Programme, Faculty of Health Sciences, Universiti Kebangsaan Malaysia, Kuala Lumpur, Malaysia; ^4^School of Biological Sciences, Universiti Sains Malaysia, George Town, Malaysia; ^5^Faculty of Chemical Engineering Technology, Universiti Malaysia Perlis, Arau, Malaysia; ^6^Centre for Chemical Biology, Universiti Sains Malaysia, Bayan Lepas, Malaysia

**Keywords:** antimicrobial resistance, antimicrobial usage, OneHealth, Malaysia, misuse

## Abstract

On a global scale, antimicrobial resistance (AMR) is recognized as a One Health challenge due to the continual and increased development and distribution of resistant microbes and genes among humans, animals, and the environment. These sectors contribute to the increase in AMR, as antibiotics are widely used in healthcare to treat or prevent bacterial infection; as growth enhancers, therapeutics and metaphylactics in animal husbandry; and transmitted in the environment through irrigation using wastewater or inappropriate disposal and treatment of human and agricultural waste. However, there is a major drawback in terms of the lack of research assessing the coexistence of AMR in these sectors. Extensive research highlighted food–animal manufacture structures that are likely to harbor reservoirs or promote transmission of AMR, in addition to increasing human colonization with AMR commensal bacteria. Numerous antibiotic stewardship policies have been designed and implemented in medical practices and animal husbandry in high- and middle-income countries. However, research concentrating on high-income settings, attitudes, emotions, and beliefs on the utilization of antimicrobials remain underexplored in lower- and middle-income countries such as Malaysia. Microbiological, epidemiological, and social science exploration are required at community and farming across the One Health range to fill huge gaps in information and knowledge of AMR. Manipulating human activities and character associated with antibiotics is a multifaceted progression that includes elements like knowledge, social behavior, attitudes, approaches, social standards, socioeconomic settings, peer pressure, experiences, and biophysical environment. Therefore, understanding these aspects in the utilization of antimicrobial drugs among the different sectors is essential to develop and implement policies to curb AMR development and transmission that overarch all sectors within the One Health consortium in Malaysia.

## Introduction

Microorganisms are known as humanity’s greatest friends and also the worst foes. Since microorganisms were discovered as the cause of contamination, it acquired time to establish their valuable role in fermentation and food and on positive influences on human health (Thakur et al., 2016). Nonetheless, the impact of the microorganism as a life-threatening agent being responsible for infections and food decay is of high relevance for individuals and also for the global economy. Several measures have been implemented globally over an immense extent of time to counter these life-threatening agents (Sharma et al., 2017).

The spatial distribution of microorganisms is often expressed according to Baas Becking’s renowned theory, “everything is everywhere but the environment selects” (Baas Becking, 1934). This implies that although microorganisms retain significant dissemination potential, only the explicitly altered organisms may flourish and multiply in a particular environment or setting. Nevertheless, molecular and ecological characteristics of microbial biogeography remain unclear as the majority of the microorganisms continue to be uncultivable/unculturable.

During early civilization, numerous molds and plant extracts have been used to treat infections. Somehow, not until the late 19th century, the scientists initiated the detection of antibacterial materials. The word antibiotic was coined by Selman Waksman to define any small molecule produced by microorganisms that could hinder the growth of other microorganisms (Clardy et al., 2009; Davies and Davies, 2010). Overall, human life expectancy and quality are raised above average with the development of antimicrobials as they can conquer the management of infectious diseases (Saga and Yamaguchi, 2009; Adedeji, 2016). Hence, scientists believe that infectious diseases can be curbed before long with this remarkable accomplishment. Based on the definition by the World Health Organization (WHO), antimicrobial resistance (AMR) means “the resistance of a microorganism to an antimicrobial drug that was initially effective for the treatment of infections caused by it” (World Health Organization [WHO], 2015).

## Global Burden of Antimicrobial Resistance

While AMR is known as a public health threat, it is also a major economic concern. Antimicrobials have played a vital role in dipping the burden of diseases caused by microorganisms and aided multifaceted medical interventions such as cutting-edge surgery procedures, cancer chemotherapy, and organ transplant. These advances are now at risk due to the occurrence and spreading of microorganisms that are impervious to antimicrobials (Smith and Coast, 2013). The high cost of treating AMR affects the economy of the country. However, in current years, AMR has developed a widespread problem and hazard to public and animal health, also a major global crisis, notwithstanding their level of income. Due to the wide usage of antibiotics, AMR has evolved as a silent killer and has become an important joint priority area recognized by both national and international agencies (Sharma et al., 2017).

A combination of evidence and economic review specified that 700,000 predictable worldwide fatalities were attributable to infections caused by AMR organisms, and by 2050, it has been predicted to be at the extent of 10 million yearly. At the same time, it is also anticipated that the mortality rate from microorganism infections, where AMR is a factor, may result in declining of 2–3.5% in the global revenue national product, totaling between 60 and 100 trillion United States dollars by 2050 (O’Neill, 2014). This monetary rate may be added apparently in low- and middle-income countries (LMICs); nevertheless, generally, the actual effect of AMR on these LMICs remains indefinite (Chandy et al., 2014). The expenses linked with AMR infections are expected to surge as further development of resistance to second- and third-generation antibiotics happens, leading to circumstances where critically ill patients require supportive care and antibiotics that no longer have therapeutic efficacy (Sartelli et al., 2020). In addition, novel antibiotic development is no longer considered to be an economically prudent venture due to economic aspects and indeterminate returns on investment. Having no major discovery on a new class of antimicrobials since the late 1980s and incomplete new drugs in the pipeline, this would severely limit therapeutic options.

## Mechanisms of Antimicrobial Resistance

Interestingly, the source of the genes related to resistance has long remained unidentified. However, in recent times, there have been growing indications representing that the majority of the bacteria found in the environment are resistant to mass groups of antibiotic elements and that this environmental pool of AMR is still rising over time (Kraemer et al., 2019). Additionally, it is well documented that AMR is a naturally occurring phenomenon upon exposure to antimicrobials due to the progression of bacterial adaptation. Mechanisms of antimicrobial resistance can be divided into four categories: (1) limiting uptake of a drug, (2) modifying a drug target, (3) inactivating a drug, and (4) active drug efflux (Reygaert, 2018). However, there is a discrepancy in the type of mechanisms employed by Gram-negative and Gram-positive bacteria due to the differences in structure.

In general, pathogenic bacteria acquire resistance by integrating additional DNA through horizontal gene transfer (HGT), conjugation, transduction and transformation. These possibilities in the acquisition of resistance include insertion of mobile element insertion sequence (IS) and transposon (Tn) that may enhance the transmission of resistance genes and the gene expression (Westphal-Settele et al., 2018). Environmental bacteria are most likely the source of antibiotic resistance genes. This is supported by various reports that have indicated the presence of clinically relevant resistance genes originating from environmental bacteria (Wright, 2010; Bengtsson-Palme et al., 2018). Hence, constructive methods are essential to reduce the hazard posed by AMR genes and resistant bacteria that transpire in the environment. The present rise in the figure of metagenomes made available in public databases suggests an opening to discover and determine the global distribution of coding sequences, commonly shared phylogenetic marker genes, and genes for HGT, which include genes of clinical importance (antibiotic resistance genes) (Fondi et al., 2016).

## Usage of Antimicrobial Agents

Antimicrobial resistance is not a new concern as this worldwide health problem started back then, in the 1970s, and this has been acknowledged by the WHO as one of the primary and major public threats of the 21st century, due to its rapid and existing widespread (Prestinaci et al., 2015). It is vital to warrant the AMR spread, as this may cause many deadly infectious diseases that would lead to further severe illness and escalation in mortality.

In clinical medicine, usage, overuse, and misuse of antimicrobial drugs that resulted as an outcome of constant progression/evolution and unchecked usage of antimicrobial drugs, which drives the acceleration of this phenomenon also known as a major causative factor in human populations. Usage of generic antibiotics over an extended time generates selective pressure on bacteria, which eliminate the susceptible bacteria and allow the antibiotic-resistant bacteria to survive, proliferate, and persist (Allcock et al., 2017).

It has also been demonstrated that multiple consensus aspects, for instance, usage of high concentrations of antibiotics or biocides with heavy metals and high bacterial count, stimulate growth, expansion, and spread of AMR (Westphal-Settele et al., 2018).

## Transmission of Antimicrobial Resistance

Generally, AMR has developed a multifactorial problem that covers human and animal health, as well as the environment, and requires an integrated approach and cross-sector intercession. Is it also noted that any form of direct or indirect connection between humans and animals could prime to zoonotic diffusion of antibiotic-resistant bacterial strains from food animals to humans. These antibiotic-resistant strains originated from animals that are able to disseminate to humans also by a series of food supply, direct contact, with the animal, or through environmental routes (Lhermie et al., 2016). In addition, several researchers have suggested an association between antimicrobial usage and the presence of antimicrobial-resistant strains not only found in livestock but also in human-to-human transmission (Sharma et al., 2017).

Antimicrobials are also largely used in domestic and livestock for health and productivity. Even though the usage of antimicrobial agents as growth enhancers has been prohibited in the European Union (EU) countries, this repetition is being continued in other countries such as America and Asia (Krishnasamy et al., 2015). Similar to human medicine, antimicrobial usage in veterinary practice (although appropriate dose used), may lead to the selection of resistance-encoding genes. These strains may be simply transmitted to humans, signifying a public health threat. A pool of these strains traits from livestock animals denotes a potential hazard for their transfer to humans.

Unmethodical usage of antibiotics in agriculture industries could also cause the dissemination of antibiotic-resistant bacteria, which may incidentally also affect the ecological microbiome (Chang et al., 2015). Soil and water are polluted with unprocessed sewage water from human waste and livestock farms or aquaculture and recycled into agricultural land leading to the groundwater containing significant amounts of antibiotic remnants and resistant bacteria (Manyi-Loh et al., 2018). Recently, the WHO strongly recommends an overall decrease in usage of all different groups of medically essential antibiotics in livestock animals, especially in food producing, as well as total restraint of these antibiotics for growth promotion and disease prevention (World Health Organization [WHO], 2017). Antibiotics are only recommended for healthy animals to avoid infection if the disease has been diagnosed in other animals from the same population. The unhealthy animals would be confirmed to regulate the best effective antibiotic to fight the most likely causative bacterium. Moreover, the antibiotics administered to the animals must be designated from the list that the WHO recognized as least “critically important” to human health and not from those categorized as “highest priority critically important.” These antibiotics should be among the last-line treatment regimens that are used to treat serious infections in humans (World Health Organization [WHO], 2017).

## Antimicrobial Resistance Surveillance

Although there has been increased attention given globally, there are considerable restrictions in the understanding of the problem, dissemination, and factors of AMR. It is also important to highlight the population-based methodologies to evaluate the correlation between antimicrobial usage and AMR in humans and animals. In general, we need to fill the missing gap in our understanding of the function of foodborne microorganisms and the general health influence at the population level. Executing the observation of AMR involving food products will need a combined and cohesive observation of AMR at the hospital and community levels (Chua et al., 2021). The overall data based on observation systems both at the national and international levels that could hypothetically recognize and gather information swiftly on guide cases are essential to enhance the understanding of the influence of these dynamics. Outlining the scope of the problem is also essential for articulating and observing a vigorous reaction to AMR. Effective and estimably linked observation programs at different and various disciplinary levels could lead to contribution for whole understanding and reduce the occurrence of resistance. Population-prevalence studies are comparable to those evaluating the health impact of antimicrobial-resistant pathogens and are vital for understanding the burden of AMR (Hay et al., 2018). These may eventually aid to construct the development of preventative interventions. A well planned national-level epidemiological study is required to understand the correlation between the AMR and usage of antimicrobial (Wall, 2019). Updated and detailed strategies for AMR stewardship in the human healthcare system and also in livestock/agriculture industries are also essential. These could also aid in identifying the high-risk populations for AMR transmission and deliver particular guidance for prevention and treatment (Allcock et al., 2017).

## Treatment and Prevention of Antimicrobial Resistance

Averting the spread of infections would be the primary instance that could diminish the need for antimicrobial consumption. This, subsequently, may moderate the expansion of resistant infections. Furthermore, the determination to widen our understanding and perspective to the aspects contributing to the occurrence and dissemination of AMR could be the main contributing factor for preemptive plans that may aid to decrease the AMR burden (Aslam et al., 2018). Several methods can be incorporated to achieve the mentioned objective, including immunization, safe and clean food preparation, measurement of disease infection control, and developed waste product organization to avert the transmission of resistant microorganisms in the hospital, agricultural, community, and environmental surroundings. Equally, creating awareness among medical practitioners, farmers, and civilians is also important to prevent antimicrobial resistance.

Nevertheless, simple health intercessions, for instance, proper sanitization practices and observance to antimicrobial stewardship can be predominantly challenging to accomplish in some of the LMICs (Lopardo et al., 2011). Additionally, human resource limitations and improper health organizations and infrastructure, limit the observation, investigation, and management of AMR (Aryee and Price, 2015). Consequently, this could be an important factor to adapt AMR preventative processes to the local setting to make them attainable and operative. Rapid diagnostic test development may be used in low- and high-resource settings to promptly detect and identify bacterial infection, and the discovery of drug resistance markers would also be useful to this effort (Burnham et al., 2017).

## History of Antibiotics in Malaysia

During earlier days in Malaysia, people relied on natural products, mostly from plants to treat wounds and diseases. Plants such as *Tridax procumbens* (“Bunga Kancing Baju”), *Curcuma longa* (“Kunyit”), and *Chromolaena odorata* (“Daun Kapal Terbang”) were commonly used where the rhizomes or leaves are ground and pasted on the wound or any infection site (Aziz et al., 2011). With the advancement in technology, research, and development of medications, antibiotics have been introduced in Malaysia, and the guidelines of antibiotic use have been established in the 1980s (Lim et al., 1993), substituting traditional medications to treat many infections based on scientific evidence.

## One Health Approach—Foundation in Malaysia

Malaysia One Health approach is mainly integrated under the guidelines of the WHO and other relevant organizations. Overall, the One Health concept was established to promote an approach to achieve better public health outcomes for humans, animals, and the environment. The foundation of the One Health approach is the 3Cs; (i) communication, (ii) coordination, and (iii) collaboration between humans, animals, and environmental professionals to share their expertise in the One Health approach. Those who may be involved in this approach are the 3Ps; (i) pharmacists, (ii) physicians, and (iii) patients, besides other healthcare and epidemiology professionals. Animal health representatives who may be involved are veterinarians and agricultural workers. The professionals representing environmental expertise are ecologists and wildlife experts (Center for Disease Control and Prevention [CDC], 2018).

At the international level, the main organization involved in the One Health concept is the WHO. The WHO has collaborated with the Food and Agriculture Organization of the United Nations (FAO) and the World Organization for Animal Health (OIE). The primary objective was to promote collaborative effort in response to food safety hazards, risks from zoonoses, and other public health threats among humans, animals, and the environment besides providing risk reduction guidance (World Health Organization [WHO], 2017).

At the national level, Malaysia has published an action plan, namely, Malaysian Action Plan on Antimicrobial Resistance (MyAP-AMR) through a collaborative effort between the Ministry of Health (MOH) and Ministry of Agriculture and Agro-Based Industry (MOA) Malaysia. In this action plan, few other departments were included. On animal aspects, the Department of Veterinary Services (DVS) and National Pharmaceutical Regulatory Agency (NPRA) are involved in the One Health approach. Environmental aspects were represented by the Pharmaceutical Services Division (PSD) under MOH. Other bodies involved in the Malaysian One Health approach are the Division of Fisheries (DOF) Malaysia, Ministry of Higher Education (MOHE) with Malaysia One Health University Network (MyOHUN), Medical Development Department, Health Education Communication Centre, Institute of Medical Research (IMR), Food Safety and Quality Division, and many more (Ministry of Health Malaysia. [MOH], 2017; Chua et al., 2021).

## Human Health—Antimicrobial Resistance in Malaysia

The Malaysian MOH has emphasized the AMR in human health and initiated numerous plans and strategies to control and restrain the dissemination, reduce the risk of AMR in human health, and govern the usage of antibiotics. Among the activities includes the establishment of the “National Surveillance of Antibiotic Resistance (NSAR)” program in the government and tertiary teaching hospitals. The NSAR was initiated in 1988, and the program was established in 2000 with the IMR as its coordinating reference laboratory (Ministry of Health Malaysia [MOH], 2018).

**FIGURE 1:**
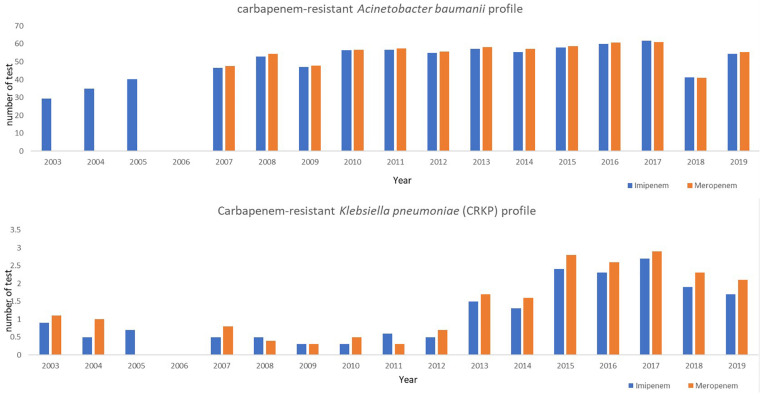
Carbapenem-resistant *Acinetobacter baumanii* and *Klebsiella pneumoniae* (CRKP) antibiotic resistance profile (NSAR 2003–2019).

Based on the first report from NSAR (2003), imipenem- and meropenem*-*resistant *Acinetobacter baumannii* was lower than 30% (Institute of Medical Research [IMR], 2020). However, there was a steep increase in the resistance rates for imipenem and meropenem to 54.4–55.5%, respectively, by 2019. Noticeably, according to the MyAP-AMR 2017–2021, *A. baumanii* resistance to meropenem has increased from 48 in 2007 to 61% by 2016. Similarly, imipenem-resistant *A. baumannii* increased from 47% in 2007 to 60% by 2016 (Figure 1; Institute of Medical Research [IMR], 2020). Additionally, a higher resistance of imipenem and meropenem was observed from intensive care units (ICUs) in several hospitals in Malaysia with mostly 70–98% (Farahiyah et al., 2017; Ministry of Health Malaysia [MOH], 2018).

At the same time, the emergence of extended-spectrum beta-lactamase (ESBL) producers among enterobacteriaceae has become a major concern in hospitals, as the ESBL-producing bacteria are often also resistant to other classes of antibiotics. *Klebsiella pneumoniae*, one of the most common causes of nosocomial infections in hospitals, has shown a slight increase in resistance toward cefotaxime from 22% in 2008 to 27.7% in 2015 (Ministry of Health Malaysia [MOH], 2018). In 2010, the New Delhi metallo-β-lactamase-1 (NMD-1) gene was first discovered in carbapenem-resistant *K. pneumoniae* (CRKP). Carbapenems such as imipenem and meropenem are considered as one of the “final course of action antibiotics” and are often used to treat serious infections caused by ESBL-carrying pathogens. The first CRKP in Malaysia was reported in 2004, an imipenem-resistant strain isolated from the blood culture of a patient. The spread of this resistance gene is swift and has resulted in reports of CRKP in various hospitals in Malaysia. CRKP (resistance to meropenem) has shown an incredible rise from 0.3% (2011) to 1.7% (2013) and reached 2.9% in 2017. Likewise, CRKP has shown an implausible rise from 0.3% (2009) to 1.5% (2013) and reached 2.7% in 2017. However, by 2019, there was a slight decrease in CRKP to 1.7 and 2.1%, respectively, (Institute of Medical Research [IMR], 2020; Figure 1).

An increase in antibiotic resistance is also commonly seen among foodborne pathogens, especially *Salmonella* spp. (non-typhoidal Salmonella), with resistance rates against ciprofloxacin and ampicillin rising to 3.4 and 25%, respectively, in 2016 (Ministry of Health Malaysia [MOH], 2018).

In 2017, the most common cause of community-acquired pneumonia, *Streptococcus pneumoniae*, was 36% resistant to erythromycin (one of the most common antibiotics used in primary care to treat respiratory tract infections). From 2013 to 2019, *S. pneumoniae* strain resistance to penicillin has been recorded between 0 to 2.3%. However, more than 31% of the *S. pneumoniae* were found to be resistant to erythromycin. Additionally, a decrease in vancomycin resistance (from 0.2 to 0.1%) was observed in 2019 compared with the increasing trend observed in 2018, wherein in 2015–2017, there was no vancomycin resistance detected (Ministry of Health Malaysia [MOH], 2019). In addition, surveillance of carbapenem-resistant enterobacteriaceae (CRE) was carried out to monitor its spread in Malaysian hospitals, which reported an alarming increase in the number of cases from 28 in 2011 to more than 800 in 2016. Based on the analysis, it showed that 95% of the patients have a history of antibiotic exposure, and of which, 50.6% had a history of antibiotic exposure for more than 7 days. Among the CRE cases, polymyxin resistance has also been reported and associated with 21.7% of deaths in the year 2016 (Ministry of Health Malaysia [MOH], 2018).

In general, based on AMR surveillance data retrieved from the years 2005, 2010, and 2019, many organisms showed a reducing trend of resistance including methicillin-resistant *Staphylococcus aureus* (MRSA) (erythromycin, gentamicin) and *Pseudomonas aeruginosa* (ceftazidime, imipenem). Alarmingly, several important Gram-negative and Gram-positive organisms such as MRSA (clindamycin), *S. pneumoniae* (erythromycin), *A. baumannii* (amikacin), *Escherichia coli* (cefuroxime, ceftazidime, ciprofloxacin), and *Salmonella* spp. (tetracycline) were continuously showing an increasing trend of AMR (Institute of Medical Research [IMR], 2020; Table 1). Thus, we can confidently presume that the effectiveness of antibiotics is collapsing, and if these persist without any strategic intervention, many of the infectious diseases that the country succeeded to curb in the past may occur again. Consequently, continuous surveillance of antibiotic usage is very important as it provides insights into the misuse of antibiotics and also aids in evaluating the impact of resistance containment interventions.

**TABLE 1:** Antimicrobial resistance of Gram-negative and Gram-positive organisms causing human infections in Malaysia (National Surveillance of Antibiotics Resistance [NSAR] 2005, 2010, and 2019; Institute of Medical Research [IMR], 2020).

Antibiotic	Organism	2005 (%) (n)	2010 (%) (n)	2019 (%) (n)
**Gram positive**				

Ciprofloxacin	*Enterococcus* sp.	40.0 (721)	32.7^#^ (490)	-
Ceftriaxone	*Streptococcus pneumoniae*	1.5 (400)	-	0.7 (143)
Clindamycin	Methicillin-resistant *Staphylococcus aureus* (MRSA)*	16.7 (1,655)	27.9 (3,159)	37.3 (5,602)
Erythromycin	Methicillin-resistant *Staphylococcus aureus* (MRSA) Streptococcus pneumoniae*	90.4 (3,797) 21.8 (481)	81.1 (3,943) 30.9 (922)	64.6 (5,953) 31.7 (1,895)
Gentamicin	Methicillin-resistant *Staphylococcus aureus* (MRSA)	89.8 (3,726)	77.2 (3,938)	10.6 (5,772)
Rifampicin	Methicillin-resistant *Staphylococcus aureus* (MRSA)	13.8 (3,816)	9.6 (3,908)	2.3 (5,636)
Vancomycin	*Streptococcus pneumoniae* *Enterococcus* sp.	0.2 (439) 1.8 (1,426)	0.0 (924) 1.2^#^ (1,207)	0.1 (1,826) 2.0^#^ (2,185)
Penicillin	*Streptococcus pneumoniae*	15 (253)	37.0^+^ (792)	0.9 (219)

**Gram negative**				

Amikacin	*Acinetobacter baumannii* * *Klebsiella pneumoniae* *Pseudomonas aeruginosa*	19.3 (3,255) 7.9 (7,873) 9.0 (10,382)	48.2∼ (9,238) 2.5 (12,248) 4.9 (12,279)	44.3 (7,495) 1.8 (28,497) 4.1 (23,174)
Ampicillin	*Salmonella* spp.	26.5 (691)	18.1 (1,361)	11.9 (1,039)
Amoxicillin	*Escherichia coli* *Klebsiella pneumoniae*	21.0 (11,402) 21.0 (9,936)	19.0 (15,119) 22.7 (13,089)	15.2 (30,076) 20.1 (30,470)
Cefuroxime	*Escherichia coli* * *Klebsiella pneumoniae*	16.0 (7,471) 27.1 (6,083)	17.3 (14,589) 21.7 (11,911)	24.5 (30,798) 25.1 (31,104)
Ceftriaxone	*Salmonella* spp.	3.1 (511)	2.7 (1,349)	0.6 (1,007)
Ceftazidime	*Acinetobacter baumannii* *Escherichia coli* * *Klebsiella pneumoniae* *Pseudomonas aeruginosa*	48.3 (3,409) 9.3 (11,364) 19.3 (10,406) 15.0 (10,325)	55.7∼ (9,614) 11.3 (15,078) 18.7 (12,177) 9.4 (13,367)	52.2 (7,337) 14.6 (30,539) 19.8 (30,510) 7.4 (22,861)
Chloramphenicol	*Salmonella* spp.	6.3 (666)	10.5 (1,171)	3.0 (724)
Ciprofloxacin	*Acinetobacter baumannii* *Escherichia coli* * *Burkholderia pseudomallei*	36.2 (3,064) 16.9 (10,850) 14.8 (155)	53.9∼ (9,482) 21.2 (15,184) 7.9 (278)	43.7^ (1,391) 24.9 (30,766) -
Gentamicin	*Acinetobacter baumannii* *Escherichia coli* *Klebsiella pneumoniae* *Pseudomonas aeruginosa*	43.6 (3,282) 14.5 (10,963) 17.5 (9,876) 18.8 (9,971)	50.6∼ (9,540) 11.2 (15,996) 14.3 (13,066) 8.2 (13,120)	47.7 (7,513) 15.3 (30,334) 8.5 (30,801) 5.5 (23,280)
Imipenem	*Acinetobacter baumannii* *Escherichia coli* *Klebsiella pneumoniae* *Pseudomonas aeruginosa*	40.3 (3,323) 0.6 (11,496) 0.7 (10,427) 11.1 (10,322)	56.5∼ (9,351) 0.4 (14,573) 0.3 (11,746) 8.1 (12,511)	54.4 (7,552) 0.5 (30,822) 1.7 (31,143) 7.7 (22,748)
Tetracycline	*Salmonella* spp.*	42.9 (438)	46.1 (555)	-
Ampicillin/ Sulbactam	*Acinetobacter baumannii* *Klebsiella pneumoniae*	40.7 (3,758) 28.2 (6,700)	51.7∼ (8,834) 26.4 (6,854)	51.5 (7,642) 25.8 (23,261)

*Increasing trend.

*^+^Based on testing with oxacillin disc.*

*^#^Enterococcus faecalis.*

*∼Acinetobacter sp.*

*^Acinetobacter baumannii from blood samples.*

*Salmonella spp.—non-typhi Salmonella.*

The exploitation of antibiotic usage investigation in Malaysia has been recognized in several government hospitals since 2001, as well as a few private hospitals. Since July 2015, this was later recognized as “National Surveillance on Antibiotic Utilisation (NSAU)” and continued to the primary care setting (MOH health clinics) (Ministry of Health Malaysia [MOH], 2016). The key findings from the surveillance demonstrated that the top five antibiotics used in hospitals are cephalosporins, fluoroquinolones, carbapenems, glycopeptides, and penicillin/beta-lactamase inhibitors. The protocol on Antimicrobial Stewardship (AMS) Programme in Healthcare Facilities was published by MOH in 2014 followed by implementation of AMS in MOH hospitals and primary care settings. The purpose of AMS establishment is to introduce a coordinated systematic approach to improve the appropriate use of antimicrobials by promoting the selection of optimal antimicrobial drug regimen in people such as the right antimicrobial choice, right route of administration, right dose, right time, right duration, and minimize harm to current and future patients. This was particularly true with MOH hospitals where 1 year after the implementation of AMS, a reduction in antibiotic utilization was seen with almost all the common antibiotics and has shown a significant improvement in adherence rate toward antimicrobial authorization policy (Sing et al., 2016).

## Animal Health and Environment—Antimicrobial Resistance in Malaysia

Antimicrobials are used widely in the agricultural sectors, including veterinary and aquaculture to increase the production of the animal via enhanced animal growth and disease prevention. The benefits of antimicrobial usage on animals include (i) healthier animals, (ii) improved food safety, (iii) secure and constant food production of livestock, (iv) uninterrupted benefits to livestock manufacturers through a minimal number of dead or sick animals, (v) suppression of the possible for an immense epidemic that could prime to the death of countless animals and destructively affect human life. However, there are risks on consuming antibiotics in animals including (i) adds to bacterial resistance in animals that could lead to treatment failure, (ii) provides bacterial resistance in humans and further complicates the management of human disease, (iii) the remaining antibiotics in food production can be a prospective threat that causes serious illness (hypersensitivity and allergic reactions) (Department of Veterinary Services [DVS], 2014). The bodies responsible for animal antibiotic usage including livestock and fisheries are MOA (DVS and DOF) and MOH (NPRA). DVS is responsible for veterinary biologics and vaccination, DOF is involved in the management and development of the fisheries industry, while NPRA is responsible for veterinary drug jurisdiction (Ministry of Health Malaysia. [MOH], 2017). The Malaysian Good Agricultural Practices (MyGAP) was established in 2013 for agricultural, livestock, and aquaculture sectors to ensure quality and safety procedures and to benchmark Malaysian products against international good agriculture practice standards.

The DVS conducted a pilot study of AMR (monitoring program of antibiotic residues) in domestic chicken, wherein in 2018, a total of 39 and 101 positive *Salmonella* spp. and *E. coli* were isolated from broiler chicken farms, respectively. A sharp increase (nearly 50%) was observed in 2019, whereby 72 and 207 positive *Salmonella* spp. and *E. coli* were isolated, respectively. However, lower numbers of *Salmonella* spp. (2) and *E. coli* (38) were recovered from layer chicken farms in 2018 compared with 11 and 56, respectively, in 2019. In addition, a nearly 10% increase in ampicillin in *Salmonella* spp. and *E. coli* resistance strains were recorded from 2018 to 2019 (70–80% and 90–92%, respectively). However, there was a sharp decrease of ampicillin resistance *Salmonella* spp. isolates from 50% (2018) to 7% (2019) in the layer chickens. Similarly, tetracycline resistance *Salmonella* spp. isolates were also decreased from 63% (2018) to 17% (2019).

A similar surveillance was also conducted in the chicken and pork slaughterhouse, where a major increase in *E. coli* was noted in chicken meat and pork in the year 2019 (106 and 24, respectively) compared with 2018 (10 and 92, respectively). In general, a vast surge was observed in the antibiotic resistance to ampicillin, chloramphenicol, and tetracycline in the slaughterhouse in 2018–2019 except for *E. coli* isolated from the chicken meat (Department of Veterinary Service [DVS], 2020). Overall, a variety of antimicrobial resistance spectrums including multidrug resistant (MDR) phenotypes among the *Salmonella* spp. and *E. coli* isolates were identified. Increased understanding of the impact of AMR on livestock and food production has further raised food security and public health concerns (Department of Veterinary Services [DVS], 2014; Table 2). In addition, a guideline for organic chicken production was established in 2014 to guide farmers to ensure that the product meets the requirements of organic chicken. One of the guidelines is no antibiotics, coccidiostatics, and any substance for growth promotion or production should be used in animal feed (Department of Veterinary Service [DVS], 2020).

**TABLE 2:** Antimicrobial resistance surveillance of *Salmonella* spp. and *Escherichia coli* in chicken farms and slaughterhouses (2018–2019) (Department of Veterinary Service [DVS], 2020).

	2018				2019			

Chicken farm	*Salmonella* spp.		*E. coli*		*Salmonella* spp.		*E. coli*	
	Broiler	Layer	Broiler	Layer	Broiler	Layer	Broiler	Layer
Number (prevalence %)	39 (15.5)	2 (3.4)	101 (39.1)	38 (65.5)	72 (30.3)	11 (22.0)	207 (87.0)	56 (50.9)
Ampicillin (% R)	70	50	90	72	80	7	92	61
Chloramphenicol (% R)	58	25	83	28	61	7	80	32
Tetracycline (% R)	84	63	91	71	79	17	94	78

	**2018**				**2019**			

**Slaughterhouse**	***Salmonella* spp. (Prevalence %)**		***E. coli* (Prevalence %)**		***Salmonella* spp. (Prevalence %)**		***E. coli* (Prevalence %)**	
	**Broiler**	**Pork**	**Broiler**	**Pork**	**Broiler**	**Pork**	**Broiler**	**Pork**

Number (prevalence %)	48 (8.90)	11 (3.31)	10 (1.86)	24 (7.23)	69 (6.86)	22 (4.80)	106 (10.54)	92 (20.09)
Ampicillin (% R)	9	63	100	29	61	77	83	57
Chloramphenicol (% R)	27	25	100	13	36	59	82	36
Tetracycline (% R)	9	71	100	46	87	96	89	36

Currently, veterinary biologics and vaccines are registered under the DVS Services Malaysia, while veterinary drugs are under the authority of the NPRA, MOH. In accordance with the Feed Act 2009, premixed antibiotics cocktail, which is used for disease prevention and growth promotion, is under the regulations of the DVS. Rendering to Registration Guideline of Veterinary Products (REGOVP), all livestock products should be listed under the NPRA, and features of quality, safety, and antibiotics residues and efficiency must be fulfilled in compliance with related legislation [Dangerous Drugs Act 1952, Poisons Act 1952, Medicine (Advertisement and Sales) Act 1956 and other related acts]. Following that, the livestock products would be verified with a series of testing and adapt to other guidelines leading to several components including manufacturing, labeling, and packaging, quality control, residual limit, and list of ingredients that are not permitted in veterinary products before the products are made accessible in the market (Hassali et al., 2018). Based on the list of registered veterinary products by the NPRA, 458 antibiotics (66.6%) out of 688 registered veterinary products have been registered for use in livestock. It is noticeable that some of those registered antibiotics are listed under the WHO’s Critically Important Antimicrobials (CIAs), where these antibiotics have been recognized as systematically vital for human health, and the use of these antibiotics should be constrained in the livestock (Table 3; Ministry of Health Malaysia. [MOH], 2017).

**TABLE 3:** Comparison of the human and veterinary critically important antimicrobials (CIAs) list (FAO/WHO/OIE, 2017).

Critically important antimicrobials	Human medicine	Veterinary medicine
Aminoglycosides	**√**	**√**
Cephalosporins (third and fourth generation)	**√**	
Macrolides	**√**	**√**
Penicillins	**√**	**√**
Quinolones	**√**	**√**
Tetracyclines	**√**	**√**
Ansamycins	**√**	
Carbapenems	**√**	
Glycopeptides	**√**	
Oxazolidinones	**√**	
Streptogramins	**√**	
Drugs used solely to treat tuberculosis or other mycobacterial diseases	**√**	
Phenicols		**√**
Sulfonamides		**√**

In addition, several antibiotics have been identified as antibiotics that are prohibited to be used in livestock due to concern about the severe harmfulness to humans and the occurrence of antibiotic-resistant strains. Those antibiotics that are not allowed or banned to be used in the livestock and aquaculture products in Malaysia include avoparcin, chloramphenicol, nitrofurans (nitrofurantoin, nitrofurazone, furazolidone, and furaltadone), teicoplanin, vancomycin, and norfloxacin (Ministry of Health Malaysia. [MOH], 2017). Avoparcin and teicoplanin share a similar activity range to vancomycin, and therefore, the usage of these antibiotics is banned to control the occurrence of vancomycin-resistant enterococci (VRE). Due to concern that could cause detrimental effects to human health, the usage of chloramphenicol and nitrofurans are banned for all livestock, whereas nitrofurans have demonstrated various toxic effects on humans (mutagenic, genotoxic, and carcinogenic properties), and chloramphenicol is suggested to lead to blood diseases including bone marrow suppression and aplastic anemia. In addition, based on the surveillance in 2018–2019, four more antibiotics have been banned by the DVS due to high resistance rate, including erythromycin, enrofloxacin, tylosin, and fosfomycin (Department of Veterinary Service [DVS], 2020).

Moreover, AMR issues involving fish production also need to be addressed; however, due to the different dimensions of aquatic biology, a particular approach needs to be constructed in combating antimicrobial resistance. AMR monitoring is relatively new in fisheries, and although there is a comprehensive ongoing monitoring program for the veterinary drug from the aquaculture farms, there has been limited research and monitoring programs for the AMR aspect. A majority of the studies focused on the prevalence of AMR pathogens, resistance patterns, and detection of resistant genes. However, there was no validation of the clinical significance of the AMR reported, and no linkages between antimicrobial use and the resistance frequencies were observed (Department of Veterinary Services [DVS], 2014). Currently, an assumption on the level of AMR in aquaculture in Malaysia could not be evaluated justifiably, and therefore, data on the usage of antimicrobials in the aquaculture sector is also restricted. According to MyAP-AMR (2017–2021), at present, no register or list of the permitted antibiotics to be used by the aqua-culturist in Malaysia is available. Based on the previous studies conducted in Asian countries, oxytetracycline, tetracycline, quinolone, sulfonamides, oxolinic acid, and trimethoprim are among the antibiotics that are allowed and commonly used (Thiang et al., 2021). Since the antibiotics used in aquaculture belong to the same group as those used for humans, the issue of AMR in fish should be equally attended to as per the One Health agenda.

In addition, the usage of antibiotics also disrupts the environmental microbiome and terrestrial. Water is being contaminated with the unprocessed effluents from human waste, livestock farms, or aquaculture may be cast off into agricultural land causing the groundwater to encompass substantial quantities of antibiotic fragments and antibiotic-resistant bacteria (Ministry of Health Malaysia. [MOH], 2017). AMR is a multifactorial issue that cuts across human and animal health as well as the environment and necessitates an amalgamated approach and cross-sector intercession. The Malaysian government has planned to strengthen AMR surveillance and monitoring, conduct more research, as well as educate and give awareness to the farmers, public, and users of antimicrobials in the animal health sector. However, this would not be feasible without a proper lab setting or expertise to address these concerns. Therefore, to avoid antibiotic contamination in the environment, PSD under MOH, Malaysia introduced the “Return Your Medicines Program” in 2010 where patients are encouraged to return their expired or unused medicine for proper disposal. The Poisons Act 1952 also recommended the development of regulations on drug disposal methods, especially for psychotropic drugs. The drugs must be incinerated to prevent contamination to the environment (Ministry of Health Malaysia. [MOH], 2017).

## Impact of Antimicrobial Resistance in Malaysia

In general, the emergence of antibiotic resistance affects aspects such as the healthcare system, public health, food security, and economy. Ultimately, humans as end users would be highly affected due to the increase in AMR across all the different sectors. The major impacts include higher medical costs, prolonged hospital stays, and increased mortality.

*Healthcare system:* The emergence of antibiotic resistance may have a direct impact on the healthcare system. It causes the number of infections to increase as the number of existing infections is added with new infections caused by resistant bacterial strains. Besides, antibiotic resistance interferes with hospital activities and limits treatment options. Some surgical procedures like bone marrow transplantation and treatment like chemotherapy may not have been opted even though they are life saving if preliminary tests revealed that a person is positive or infected with resistant bacteria since it may give rise to complications afterward. As the choices of antibiotics are limited, and the guidelines are constantly updated, in Malaysia, compliance to the use of the latest approved antibiotics may be compromised especially in the rural areas.

*Public health:* Antibiotic resistance not only affects those who are non-infected but also those who are not infected with antibiotic-resistant bacteria. The empirical or narrow-spectrum antibiotic choices are also limited for all patients regardless of their resistance status. The regulatory bodies frequently update and change the guideline on antibiotic usage and prescription according to the latest antibiotic-resistant update and discoveries. The higher the number of antibiotic resistant discovered, the more limited the choices of antibiotics to be used for infection control, and the remaining available choices may be highly toxic. Antibiotic resistance may also increase the morbidity and mortality of the patients as the infection can be more severe and harder to treat (Ahmad, 2019). Individuals hospitalized without bacterial infections may acquire MRSA infections due to prolonged hospital stays. Of all in-patients with MRSA infection, 86.3% have acquired the infection in the hospital, and 11.76% have succumbed to septicemia secondary to MRSA infection (Abidin et al., 2020).

*Food security (agriculture and aquaculture):* Antibiotics have been used in animal feed as an infection prophylaxis and growth promoter. This has contributed to the emergence of antibiotic resistance. Some countries have banned the usage of antibiotics on livestock and in agro-industrial plants; however, many other countries have yet to set a legislation on antibiotic usage in these industries the reason being consumer demands are too high; therefore, without this enhancer, their production could not meet consumer demand, hence, reducing their turnover. The MOH in collaboration with the MOA and Agro-based Industry during National Antibiotic Awareness Week Campaign in November 2019 has considered expanding the list of antibiotics to be banned for growth promoter after banning colistin in animal feeds in 2018 (Ministry of Health Malaysia [MOH], 2019).

*Economy:* Resistant bacterial infections are more likely to cause an economic burden as the expenses per infected patient increase. A person with resistant bacterial infection may require longer hospital or ICU stay for prolonged and more severe illness with extra diagnostic tests, nursing, and medical care. It was reported that the hospital expenses for each resistant bacterial infection would at least double compared with normal infection (Ahmad, 2019). Besides that, the antibiotics used to replace those bacteria are resistant to, are usually more expensive. Malaysia spent around RM 165 million to buy antibiotics for all government hospitals and primary care clinics each year. To reduce the economic burden of antibiotics, the usage of antibiotics must be frequently monitored (Shamsuddin et al., 2016).

## Challenges and Outcome of One Health Toward Antimicrobial Resistance

The major challenges faced in the One Health approach in the Malaysian context are the lack of surveillance, the lack of monitoring, and the lack of baseline data, such as antibiotic usage, registration, emergence, and awareness on the importance to control the development and spread of AMR, especially in the animal and fisheries sector. Although scarce, various pieces of evidence suggest the existence of resistance in all the different sectors. To a certain extent, data from human healthcare and animal husbandry are more established. Thus, there is an urgent need to establish this baseline data as a driver to further planning and action to curb the development of AMR. Involvement and high commitment of various agencies including the MOH, DVS, DOF, and NPRA coupled with combined efforts may ensure a successful outcome. Currently, there is awareness on these aspects, and combined efforts are initiated in order to establish relevant policies and activities that can help to tackle the rise of AMR. Table 4 shows the summary of the different organizations involved and the policies that are being implemented for this purpose.

**TABLE 4:** Malaysian One Health implementation programs.

Program/department/organization	Objective	Policies/activities
**Human**		

Antimicrobial Stewardship Program by Ministry of Health Malaysia [MOH] (2014)	To introduce a systematic approach to improve appropriate antimicrobial use	• Indication explicitly spelt out • Investigate before prescribe • Follow National Antibiotic Guideline • Establish a list of restricted antimicrobials • Restrict broad-spectrum AB use • Consider progress of patient

**Animal (agriculture and food security)**		

Department of Veterinary Services Malaysia (DVS) and Department of Fisheries (DOF)—Animal Feed Act 2009/Food Safety and Quality Division National Pharmaceutical Regulatory Agency (NPRA), MOH	To prevent and control animal and zoonotic diseases, to ensure food of animal origin is clean and fit for human consumption, to promote the growth and development of the animal feed industry, and to ensure the welfare and wellbeing of all animals To ensure that therapeutic substances approved for the local market are safe, effective, and of quality and also to ensure that cosmetic products approved are safe and of quality	• Register veterinary products to NPRA • Monitoring program of AB residues by DVS • Malaysian Good Agricultural Practices (MyGAP) 2013 • Organic chicken production guideline (2014)

**Humans and animal**		

The Malaysia One Health University Network (MyOHUN) (under MOHE) was established in 2012 as a regional network of Southeast Asia One Health University Network (SEAOHUN)	To forge collaborations between academicians, professionals, scientists, and communities with responsibility for human and animal health	• Seminar and workshop on infectious and zoonotic diseases • Training trainers on AMR • Infectious disease simulation exercise • Community education • AMR educational toolkit development

**Environment**		

“Return Your Medicines” Program by PSD in 2010 Guideline for drug disposal by PSD under the Poisons Act 1952	To encourage patients to return unused or expired medicines Drugs disposed by incineration to prevent environmental pollution	• Medicines that are no longer needed can be returned to the pharmacy counter or medicine return boxes provided at all pharmacy facilities in MOH hospitals and health clinics

Although various policies and relevant acts exist to aid regulation and usage of antibiotics in healthcare and animal feed, irresponsible use, poor monitoring, and enforcement of regulations in Malaysia may pose a major challenge to the One Health approach. Various measures can be taken to overcome these challenges, where the government, general public, healthcare personnel, and farmers have to come together and play their roles. Education of the community is essential to increase knowledge and understanding of AMR as well as to create awareness and a sense of responsibility. This should be emphasized more in people who are involved in healthcare, food, and livestock production as well as aquaculture. The establishment of infection control policies and antibiotic stewardship in the healthcare setting may help in reducing the development of AMR. In addition, continuous AMR surveillance in all different sectors is also important to monitor the emergence of AMR. As much as it is important to establish policies and action plans associated with the control and prevention of AMR, its enforcement is equally important.

## Conclusion and Future Perspective

Antimicrobial resistance is a worldwide public health threat, and it is noticeable that despite rising worldwide attention to AMR, there are significant restrictions and limitations in our understanding of the AMR burden, dissemination, and determinants. It is important to bring together different research communities including microbiologists, epidemiologists, veterinarians, and social science researchers to obtain new sources of data to fill the large gaps in knowledge and to develop appropriate intervention strategies of AMR across the OneHealth spectrum. Additionally, this also may improve our understanding of the AMR burden in populations, as well as the population-level aspects prompting the expansion and spreading of AMR in Malaysia. The current situation of AMR in Malaysia is still uncertain and, based on the present research in this field, has not been extensively connected to the public. The strategy underlining the disease prevention approaches, as well as enhancement of biosecurity and farming, surge in the use of vaccines, and consolidation of observation composed of educational and informative campaigns and awareness of AMR should be addressed in all levels of society. However, several research gaps need to be highlighted: (i) Is there a widespread occurrence of an antibiotic-resistant gene in the population, livestock, and environment?, (ii) Do dietary practices, lifestyle habits, or agricultural practices affect the antibiotic-resistant gene reservoir in a particular geographical region?, and (iii) What and how do these behavioral traits contribute to the dissemination of antimicrobial resistance in the population?

## Author Contributions

VM, KV, and JV conceptualized the manuscript. VM, KV, and NM wrote the manuscript. SS and JV revised and edited the manuscript. All authors have critically reviewed the manuscript.

## Conflict of Interest

The authors declare that the research was conducted in the absence of any commercial or financial relationships that could be construed as a potential conflict of interest.

## Publisher’s Note

All claims expressed in this article are solely those of the authors and do not necessarily represent those of their affiliated organizations, or those of the publisher, the editors and the reviewers. Any product that may be evaluated in this article, or claim that may be made by its manufacturer, is not guaranteed or endorsed by the publisher.
